# Antiretroviral therapy abandonment among adolescents and young people with HIV/AIDS during COVID-19: A case-control study*

**DOI:** 10.1590/1518-8345.6497.3948

**Published:** 2023-06-19

**Authors:** Camila Moraes Garollo Piran, Alana Vitória Escritori Cargnin, Bianca Machado Cruz Shibukawa, Natan Nascimento de Oliveira, Marcelo da Silva, Marcela Demitto Furtado

**Affiliations:** 1 Universidade Estadual de Maringá, Maringá, PR, Brasil.; 2 Prefeitura do Município de Maringá, Ambulatório Municipal de IST/HIV/AIDS, Maringá, PR, Brasil.

**Keywords:** HIV, Acquired Immunodeficiency Syndrome, COVID-19, Patient Dropouts, Adolescent, Young Adult, VIH, Síndrome de Inmunodeficiencia Adquirida, COVID-19, Pacientes que Abandonan el Tratamiento, Adolescente, Adulto Joven, HIV, Síndrome de Imunodeficiência Adquirida, COVID-19, Pacientes Desistentes do Tratamento, Adolescente, Adulto Jovem

## Abstract

**Objective:**

to identify the factors associated with antiretroviral therapy abandonment among adolescents and young people living with HIV/AIDS during the COVID-19 pandemic.

**Method:**

a case-control study carried out between 2020 and 2021 in Maringá, Paraná. The cases corresponded to the following: adolescents and young people (aged from 10 to 24 years old) diagnosed with HIV/AIDS and who abandoned treatment, while the Control Group consisted of people with similar sociodemographic characteristics, diagnosed with HIV/AIDS and with no history of treatment abandonment. Pairing of the cases and controls was by convenience, with four controls for each case. The research instrument presented sociodemographic variables, clinical characteristics and others, whose association with treatment abandonment was analyzed by means of logistic regression.

**Results:**

a total of 27 cases and 109 controls were included in the study (1/4 ratio). The variable associated with an increased chance of abandonment was age close to 22.8 years old (OR_adj_: 1.47; 95% CI: 1.07-2.13; p=0.024). Sporadic condom use (OR_adj_: 0.22; 95% CI: 0.07-0.59; p=0.003) and having an opportunistic infection (OR: 0.31; 95% CI: 0.10-0.90; p=0.030) were protective factors.

**Conclusion:**

age close to 23 years old at the last consultation was associated with antiretroviral therapy abandonment. The presence of opportunistic infections and condom use are determining factors for treatment continuity during COVID-19.

## Abstract (es)

**Objetivo:**

identificar los factores asociados al abandono de la terapia antirretroviral entre adolescentes y jóvenes que vivían con VIH/sida durante la pandemia de COVID-19.

**Método:**

estudio de casos y controles realizado entre 2020 y 2021 en Maringá, Paraná. Los casos fueron: adolescentes y jóvenes (10 a 24 años) diagnosticados con VIH/sida y que abandonaron el tratamiento, mientras que el grupo de controles fue compuesto por personas con características sociodemográficas semejantes, diagnosticadas con VIH/sida, sin historia de abandono del tratamiento. El emparejamiento de casos y controles se hizo por conveniencia, con cuatro controles por cada caso. El instrumento de investigación presentó variables sociodemográficas y clínicas, entre otras y la asociación con el abandono del tratamiento se analizó por regresión logística.

**Resultados:**

se incluyeron 27 casos y 109 controles en el estudio (proporción 1/4). La variable asociada con mayor probabilidad de abandono fue la edad próxima a 22,8 años (OR_aj_:1,47; IC 95%:1,07-2,13; p=0,024). El uso esporádico del preservativo (OR_aj_:0,22; IC 95%:0,07-0,59; p=0,003) y padecer una infección oportunista (OR:0,31; IC 95%:0,10-0,90; p=0,030) fueron factores protectores.

**Conclusión:**

la edad próxima a los 23 años en la última visita se asoció al abandono de la terapia antirretroviral. La presencia de infección oportunista y el uso de preservativos son factores determinantes para la continuidad del tratamiento durante el COVID-19.

## Abstract (pt)

**Objetivo:**

identificar os fatores associados ao abandono da terapia antirretroviral entre adolescentes e jovens vivendo com HIV/aids durante a pandemia de COVID-19.

**Método:**

estudo caso-controle realizado entre 2020 e 2021 em Maringá, Paraná. Os casos foram: adolescentes e jovens (10 a 24 anos) diagnosticados com HIV/aids e que abandonaram o tratamento, enquanto o grupo dos controles foi composto por pessoas com características sociodemográficas semelhantes, diagnosticadas com HIV/aids, sem histórico de abandono de tratamento. O pareamento dos casos e controles foi por meio de conveniência, sendo quatro controles para cada caso. O instrumento de pesquisa apresentou variáveis sociodemográficas, características clínicas e outras, cuja associação com o abandono do tratamento foi analisada por meio de regressão logística.

**Resultados:**

27 casos e 109 controles foram incluídos no estudo (proporção 1/4). A variável associada à maior chance de abandono foi idade próxima de 22,8 anos (OR_aj_.:1,47; IC95%:1,07-2,13; p=0,024). O uso esporádico de preservativo (OR_aj_:0,22; IC95%:0,07-0,59; p=0,003) e ter infecção oportunista (OR:0,31; IC95%:0,10-0,90; p=0,030) foram fatores de proteção.

**Conclusão:**

idade próxima a 23 anos na última consulta foi associada ao abandono da terapia antirretroviral. A presença de infecção oportunista e o uso de preservativo são fatores determinantes para continuidade do tratamento durante a COVID-19.

**Highlights:**

**(1)** Age close to 22.8 years old increases the chances of antiretroviral therapy abandonment.

**(2)** The distance to the service is a control variable that favors abandonment.

**(3)** Sporadic condom use and opportunistic infections are protective factors.

**(4)** Adolescents and young people have more vulnerable behaviors.

**(5)** Reflections on antiretroviral therapy abandonment during COVID-19.

## Introduction

At the end of 2019, in the city of Wuhan, China, beginning of transmission of the SARS-CoV-2 virus was observed, the etiological agent causing the Severe Acute Respiratory Syndrome 2, known as COVID-19. Then, the pandemic state was declared in March 2020, becoming one of the greatest health challenges today^(1)^.

People with chronic comorbidities such as diabetes, hypertension, asthma, cardiovascular disease, obesity, chronic lung disease, liver disease and kidney disease and immunocompromised people, among which those living with the Human Immunodeficiency Virus (HIV) stand out, were advised to adopt extra precautions, as they are more susceptible to serious conditions and complications due to COVID-19 and, consequently, higher risks of death^(2)^.

Even more than three decades after the emergence of HIV, the agent responsible for the Acquired Immunodeficiency Syndrome (AIDS), the disease still represents a serious challenge for public health in the world. Nearly 37.7 million people were living with HIV in 2020 and only 28.2 million had access to antiretroviral therapy (ART). In addition to that, it is estimated that approximately 5 million were young individuals between the ages of 15 and 24^(3)^.

In Brazil, in the female age group from 15 to 24 years old, there was a reduction in the HIV detection rate and, in 2010, it was 18.5 *per* 100,000 inhabitants, dropping to 9.2 per 100,000 inhabitants in 2020. In relation to males, in the same age group, the rate rose from 27.3 to 33.2 *per* 100,000 inhabitants in the 2010 and 2020 periods, respectively. In addition to that, the AIDS mortality rate also increased among young men aged from 20 to 24 years old, with a rate of 3.1 and 3.4 deaths *per* 100,000 inhabitants in 2010 and 2020, respectively^(4)^.

This period of adolescence and youth is marked by intense physical, emotional and behavioral changes, which involve new experiences, some of which can pose health risks. In view of this, the health system needs to broaden the view of the factors associated with Sexually Transmitted Infections (STIs), bringing up discussions about vulnerability, sexuality and STIs such as HIV, consequently increasing the amount of information about HIV tests, early diagnosis, proper treatment and prevention^(5-6)^.

Each individual or social group has different life styles and experiences, risks and perceptions related to HIV/AIDS and, therefore, the offer of preventive methods should be diversified and broad in order to provide greater coverage among the population. Thus, combined prevention is a very effective strategy because it involves using different prevention approaches that can be employed by several levels of society, in order to meet the needs of different population segments^(7)^.

Among the prevention strategies, it is worth noting that ART aims at reducing the viral load in individuals diagnosed with HIV/AIDS, with the possibility of even becoming undetectable and, thus, reducing the virus transmission risks, in addition to improving the quality of life and life expectancy of people living with HIV/AIDS. However, despite the existence of several intervention and prevention strategies to contain this health problem, treatment non-adherence and/or abandonment remains a global challenge, reflected in the increase in number of cases in certain age groups^(8-10)^.

For successful treatment, adherence to ART is one of the main factors involved. However, it is noticed that there is still ART discontinuation and abandonment; however, the reasons associated with this outcome are not fully known^(11-12)^, even more in the face of a pandemic period, which impacted people’s lives in different ways, both in the social sphere and in the health and disease process^(13)^.

It is noted that the data found in the literature are controversial and insufficient, as there are reports of factors associated with age, distance from the service, family income, knowledge about HIV/AIDS and opportunistic infections^(11-12)^. However, the studies found are not consistent with each other. Therefore, the authors conducted a scoping review in order to map the reasons for treatment non-adherence or abandonment among adolescents and young people living with HIV/AIDS. Such search highlighted that the state-of-the-art regarding the theme is incipient in the national and international contexts and that, in most cases, only cross-sectional and descriptive studies are carried out, which do not produce evidence with high levels of confidence, compromising advances in the area.

Therefore, this study is justified by the need to elucidate the complexity involved in the ART abandonment by means of methods that have higher levels of evidence. In turn, longitudinal studies such as this one are crucial to identify and analyze the intrinsic and extrinsic factors regarding ART abandonment, in addition to producing subsidies for advances in the health care.

The results of this study will assist health professionals, especially nurses, in the elaboration of a comprehensive care plan and ART adherence strategies according to the needs of each adolescent and young person living with HIV/AIDS It is noted that the pandemic period is permeated by challenges for patient safety care and medication availability linked to social isolation. Therefore, the objective of this study was to identify the factors associated with ART abandonment among adolescents and young people living with HIV/AIDS during the COVID-19 pandemic.

## Method

### Study design

A case-control study with a ratio of 1 case/4 controls in adolescent and young. The recommendations set forth in the Strengthening the Reporting of Observational Studies in Epidemiology (STROBE) checklist^(14)^ were followed to conduct and present the study.

### Study locus

The study locus was the Specialized Care Service (*Serviço de Atenção Especializada*, SAE) belonging to the STI/HIV/AIDS Outpatient Service of the 15^th^ Health Region, located in the municipality of Maringá-Paraná, which offers another two services: the Testing and Counseling Center (*Centro de Testagem e Aconselhamento*, CTA) and the Medication Dispensing Unit The study was carried out at this location because it is a reference service that provides care to people with STIs living in one of the 30 municipalities belonging to the 15^th^ Health Region, which are formally included in the care network.

### Participants

The cases were defined as follows: adolescents and young people (aged between 10 and 24 years old), with the following International Classification of Diseases (ICD) — serves as a basis for identifying statistical trends in mortality and morbidity worldwide — codes referring to HIV/AIDS: B20.0 to B24, with subsequent ART abandonment. The controls were defined as adolescents and young people with ICD codes related to HIV/AIDS, undergoing monitoring at the SAE, with no history of ART abandonment.

The following inclusion criteria were considered: being aged between 10 and 24 years old and living in a municipality linked to the 15^th^ Health Region of the state of Paraná. The exclusion criteria were patients who died (one), transferred to other health services (five) and medical records not identified in the service (three).

It is noted that the expressions “adolescence” and “youth” refer to the age groups from 10 to 19 and from 20 to 24 years old, respectively^(15)^.

### Sample

After an exploratory analysis of the medical records of the prospective participants, the use of the total population was defined in detriment of a sample. Thus, all SAE users were included in the sample, observing the aforementioned selection criteria. The pairing of one (1) case to four (4) controls was for convenience. Thus, four controls (109) were selected for each case (27).

**Figure 1 -:**
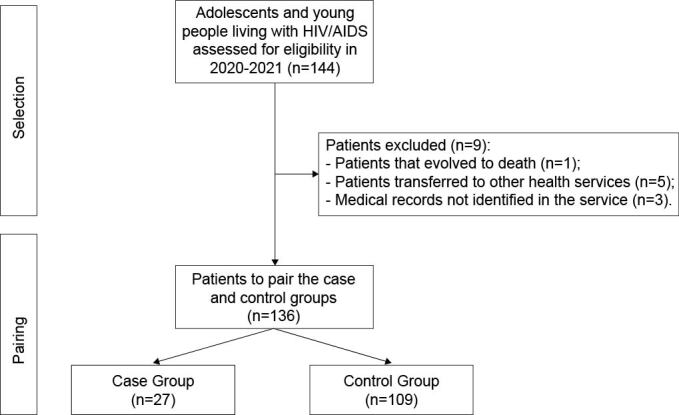
Flowchart synthesizing the selection of patients who made up the case and control groups of adolescents and young people living with HIV/AIDS in the 15^th^ Health Region. Maringá, PR, Brazil, 2022

The pairing process took place according to the participants’ sociodemographic characteristics, considered as non-modifiable intrinsic factors such as gender, age and abandonment outcome. In addition, the pairing considered the area of residence of the service users, in order to guarantee homogeneity between the groups. Figure1 presents a flowchart designed to show the selection of the patients.

The recruitment, exposure and monitoring period was between March 2020 and December 2021, being marked by the advent of the COVID-19 pandemic. Data collection was carried out between March and May 2022.

### Variables

All variables were collected from the health records. A dependent variable (ART abandonment) was adopted for the logistic regression, verifying the influence of independent variables on the dependent variable.

#### Dependent variable

ART abandonment was considered as dependent variable (outcome). The definition of ART abandonment, according to Technical Note No. 208/09 of the Ministry of Health, is not fetching antiretroviral medications from the service pharmacy, from three months after the scheduled date and not returning to the consultations in six months^(16)^.

#### Independent variables

Variables targeted at the sociodemographic characteristics [age at diagnosis, age at last consultation, marital status, sexual orientation, race/skin color, schooling, occupation, area of residence, housing, home-service distance in minutes (min) and in kilometers (km)] were selected; as well as epidemiological and behavioral characteristics (tobacco use, alcohol consumption, illicit drugs, condoms use, type of sex partner, HIV+ partner, comorbidities, diagnosis of mental disorders, use of psychotropic drugs, criminal history, living on the streets); and characteristics of diagnosis and monitoring (transmission means, referral locus, first viral load result, last viral load result, first CD4+ result, last CD4+ result). In order to ensure data comparability, the instrument was used both the cases and for the controls.

### Data source

The data were collected from secondary sources through medical records made available at the outpatient service by the main researcher. The data collection instrument was prepared based on the Ministry of Health recommendations regarding the notification form, ART and outpatient follow-up of HIV/AIDS cases. The instrument underwent content and semantic analysis during the research project evaluation process to verify whether it was suitable to be used and respond to the study objective, even though it was a collection of secondary data.

### Bias

The selection bias was addressed using the record numbers of the medical charts, which were checked by two independent researchers.

During data processing, outliers were removed so as to maintain integrity of the database and results, reducing the chance of analysis bias. In addition, the analyses considered missing values as null, removing the participants that did not present all the listed variables, mitigating the action of confounding factors.

The study had its data and results validated by specialists in the method, as a way to avoid interpretation bias. However, the possibility for the occurrence of biases and confounding factors is highlighted, being listed as study limitations.

### Data analysis

The data were tabulated in electronic spreadsheets, imported and analyzed in the R software, version 4.2.0. The descriptive analysis was conditioned to the nature of each variable; thus, the categorical variables were described by means of absolute and relative frequencies using Pearson’s Chi-Square Test and Fisher’s Exact Test, according to assumptions for the use of each test. For quantitative variables, normality was tested using the Shapiro-Wilk test and evaluation of histograms, with subsequent use of Student’s or Wilcoxon’s t tests, the parametric test and its non-parametric counterpart, respectively.

To identify the factors associated with ART abandonment, a logistic regression model was used, with the “ART abandonment or not” dependent variable classified as Abandonment (1) and as Non-abandonment (0). Each explanatory variable was tested individually and those with p-values < 0.20 were included in the initial multiple model. The stepwise methodology was also used to find the best fit of the final model and, for permanence in the final model, a 5% significance level or control of the test parameters was adopted. The stepwise methodology uses the Akaike Information Criterion (AIC) to identify the model that fits properly.

Adequacy of the regression model was evaluated based on the assumptions of heteroscedasticity and normality of the residuals, in addition to maximum likelihood tests for estimating the coefficients. To identify the possible multicollinearity of the variables, covariance was tested using covariance matrices and the Variance Inflation Factor (VIF), considering values greater than or equal to 2.5 as positive indicators.

In order to ease interpretation of the coefficients (β), exponentiation was performed to estimate the Odds Ratio (OR) and 95% Confidence Intervals (95% CIs). In addition, a predictive evaluation of the model was performed based on the estimation of the Receiver Operating Characteristic Curve (ROC Curve) and a Confusion Matrix, with accuracy, sensitivity and specificity estimations, applying the McNemar Test to identify significance, with 95% level significance.

### Ethical aspects

Although there was no direct contact with the patients, the medical records present diverse individual information that is confidential. Thus, the study was developed following all the ethical and legal precepts of Resolutions No. 466/12 and No. 510/16 of the National Health Council (*Conselho Nacional de Saúde*, CNS), being approved in the Standing Committee on Ethics in Research Involving Human Beings of the State University of Maringá/PR under opinion No. 5,202,623 (CAAE: 52331221.3.0000.0104).

### Results

A total of 144 adolescent and young patients (aged from 10 to 24 years old) living with HIV/AIDS and treated in the SAE during COVID-19 were registered, with 136 adolescents and young people eligible for the study, of which 24 were cases (Abandonment) and 109 controls (Non-abandonment). In relation to the sociodemographic profile of adolescents and young people living with HIV/AIDS from the case and control groups, 89% were diagnosed when they were between 20 and 24 years of age, male and without a partner, respectively. It was noticed that 81% of the cases and 89% of the controls were homosexual/bisexual, that 70% of the white-skinned subjects abandoned treatment and that 47.3% did not abandon ART. In both groups, 90% had up to 12 years of study, 59% were unemployed/housewives and, in addition, in the Case Group, 96% lived in the urban area whereas 98% did so in the Control Group. Therefore, these findings allow verifying how homogeneous the data of the groups are. A statistically significant association was identified between ART abandonment and the following variables: “age at last consultation”, “age close to the mean of 22.8 years old” (p=0.132) and “distance from the service to the patient’s home in km”, being 17.3 km (p=0.012) (Table1).

**Table 1 -:** Distribution of the sociodemographic characteristics corresponding to the adolescents and young people living with HIV/AIDS in the 15^th^ Health Region. Maringá, PR, Brazil, 2022

Sociodemographic characteristics	Abandonment	Non-abandonment	Total	p value*
	n=27	n=109	N=136	
	n(%)^†^	n(%)^†^	N(%)^†^	
**Age at diagnosis (years)**				0.764^‡^
15-19	3 (11.0)	17 (16.0)	20 (14.7)	
20-24	24 (89.0)	92 (84.0)	116 (85.3)	
**Age at last consultation**				0.132^§^
	22.8 (1.2)^||^	22 (2.8)^||^	22.1(2.6)^||^	
**Gender**				>0.999^‡^
Female	3 (11.0)	12 (11.0)	15 (11.0)	
Male	24 (89.0)	97 (89.0)	121 (71.3)	
**Marital status**				>0.999^‡^
No partner	24 (89.0)	97 (89.0)	121 (89.0)	
Has a partner	3 (11.0)	12 (11.0)	15 (11.0)	
**Sexual orientation**				0.330^‡^
Heterosexual	5 (19.0)	12 (11.0)	17 (12.5)	
Homosexual/Bisexual	22 (81.0)	97 (89.0)	119 (87.5)	
**Skin color/Race**				0.233^‡^
Asian	0 (0.0)	1 (1.8)	1 (1.2)	
White	19 (70.0)	26 (47.3)	45 (54.9)	
Brown	7 (26.0)	23 (41.8)	30 (36.6)	
Black	1 (3.7)	5 (9.1)	6 (7.3)	
**Schooling**				>0.999^‡^
<12 years	19 (90.0)	81 (90.0)	100 (90.1)	
12+ years	2 (9.5)	9 (10.0)	11 (9.9)	
**Occupation**				0.793^‡^
Services	5 (19.0)	16 (15.0)	21 (15.4)	
Technical/Higher Level Occupation	6 (22.0)	29 (27.0)	35 (25.7)	
Others (Unemployed/Housewife)	16 (59.0)	64 (59.0)	80 (58.8)	
**Area of residence**				0.488^‡^
Rural	1(3.7)	2(1.8)	3 (2.2)	
Urban	26(96.0)	107(98.0)	133 (97.8)	
**Housing**				0.952^¶^
Lives alone	9 (33.0)	40(37.0)	49 (36.3)	
Lives with family members/partner	15(56.0)	56(52.0)	71 (52.6)	
Lives with friends/acquaintances	3(11.0)	12(11.0)	15 (11.1)	
**Distance to the service (in km** ^**^ **)**				0.012^§^
	17.3(20)^||^	10.9(13.3)^||^	12.2(15)^||^	
**Distance to the service (in min** ^††^ **)**				0.052^§^
	66.8(33.5)^||^	56.1(38)^||^	58.2(37.3)^||^	

*Significant when <0.20; ^†^Absolute number and percentage = (The totals may differ due to the possibility of non-answer or non-completion in the patient’s medical record); ^‡^Fisher’s Exact Test; ^§^Normality test by the Shapiro-Wilk test and evaluation of histograms, with subsequent use of Student’s or Wilcoxon’s t tests, parametric test and its non-parametric counterpart; ^||^Mean (Standard Deviation); ^¶^Pearson’s Chi-Square Test; ^**^km = Kilometer; ^††^min = Minutes

It is noted that the “Distance to the service in kilometers” variable was chosen as the preferred variable to be included in the multiple model because it is a fixed distance, whereas the distance in minutes is an estimate and is influenced by other external factors. Inclusion of both variables in the model would generate multicollinearity, disabling the regression.

Among the epidemiological and behavioral characteristics, none of the adolescents or young people had a criminal record or lived on the streets during the study period. Although 60% did not drink alcohol, 62.2% used tobacco and 71.1% used other drugs. The following variables were associated with ART abandonment: tobacco use (p=0.092) and condom use (p=0.006). Among the young people who abandoned ART, there were proportionally fewer smokers and more individuals who did not always use condoms. As for condom use, only 12.5% stated always using them. In relation to the type of sex partner, 58.1% had occasional partners. The minority had comorbidities (4.4%), mental disorders (17.7%) and used psychotropic drugs (14.7%)(Table2).

**Table 2 -:** Distribution of the epidemiological and behavioral characteristics corresponding to the adolescents and young people living with HIV/AIDS in the 15^th^ Health Region. Maringá, PR, Brazil, 2022

Epidemiological and behavioral characteristics	Abandonment	Non-abandonment	Total	p value*
	n=27	n=109	N=136	
	n(%)^†^	n(%)^†^	N(%)^†^	
**Alcohol use**				0.334^‡^
Yes	13 (48.0)	41 (38.0)	54 (40.0)	
No	14 (52.0)	67 (62.0)	81 (60.0)	
**Tobacco use**				0.092^‡^
Yes	13 (48.0)	71 (66.0)	84 (62.2)	
No	14 (52.0)	37 (34.0)	51 (37.8)	
**Illicit drugs**				0.296^‡^
Yes	17 (63.0)	79 (73.0)	96 (71.1)	
No	10 (37.0)	29 (27.0)	39 (28.9)	
**Condom use**				0.006^§^
Never	15 (56.0)	29 (27.0)	44 (32.4)	
Sometimes	8 (30.0)	67 (61.0)	75 (55.1)	
Always	4 (15.0)	13 (12.0)	17 (12.5)	
**Type of sex partner**				0.463^‡^
Occasional	14 (52.0)	65 (60.0)	79 (58.1)	
Steady	13 (48.0)	44 (40.0)	57 (41.9)	
**HIV+ partner**				0.523^§^
No	2 (7.4)	17 (16.0)	19 (14.0)	
Yes	8 (30.0)	25 (23.0)	33 (24.3)	
I don’t know	17 (63.0)	67 (61.0)	84 (61.8)	
**Comorbidities**				>0.999^§^
Yes	1 (3.7)	5 (4.6)	6 (4.4)	
No	26 (96.0)	104 (95.0)	130 (95.6)	
**Diagnosis of mental disorders**				0.784^§^
Yes	4 (15.0)	20 (18.0)	24 (17.7)	
No	23 (85.0)	89 (82.0)	112 (82.3)	
**Use of psychotropic drugs**				0.764^§^
Yes	3 (11.0)	17 (16.0)	20 (14.7)	
No	24 (89.0)	92 (84.0)	116 (85.3)	

*Significant when <0.20; ^†^Absolute number and percentage = (The totals may differ due to the possibility of non-answer or non-completion in the patient’s medical record); ^‡^Pearson Chi-Square Test; ^§^Fisher’s Exact Test

As for the characteristics of diagnosis and monitoring, the “opportunistic infections”, “first viral load result” and “last CD4+ result” variables presented statistical significance (Table3).

**Table 3 -:** Distribution of the characteristics of the diagnosis, treatment and monitoring corresponding to adolescents and young people living with HIV/AIDS in the 15^th^ Health Region. Maringá, PR, Brazil, 2022

Characteristics of diagnosis and monitoring	Abandonment	Non-abandonment	Total	p value*
	n=27	n=109	N=136	
	n(%)^†^	n(%)^†^	N(%)^†^	
**Referral locus**				0.301^‡^
CTA ^§^	12 (44.0)	66 (61.0)	78 (57.4)	
Hospital/Blood Center	1 (3.7)	7 (6.4)	8 (5.9)	
Transfer from another SAE^||^	10 (37.0)	26 (24.0)	36 (26.5)	
BHU^¶^	4 (15.0)	10 (9.2)	14 (10.3)	
**Opportunistic infections**				0.065^**^
Yes	17 (63.0)	87 (80.0)	104 (76.5)	
No	10 (37.0)	22 (20.0)	32 (23.5)	
**First viral load result**				0.047^††^
	83,537.6 (244,813.8)^‡‡^	115,807.7 (196,625.6)^‡‡^	110,253.0 (204,958.0)^‡‡^	
**Last viral load result**				0.538^††^
	11,801.5 (18,348.1)^‡‡^	83,612.6 (204,373.0)^‡‡^	64,804.9 (177,948.0)^‡‡^	
**First CD4+** ^§§^ **result**				0.113^††^
	605.9 (296.3)^‡‡^	589.2 (743.4)^‡‡^	592.1 (684.9)^‡‡^	
**Last CD4+** ^§§^ **result**				0.004^††^
	1,499.2 (3,038.8)^‡‡^	1,105.6 (3,620.2)^‡‡^	1,174.2 (3,516.0)^‡‡^	

^*^Significant when <0.20; ^†^Absolute number and percentage = (The totals may differ due to the possibility of non-answer or non-completion in the patient’s medical record); ^‡^Fisher’s Exact Test; ^§^CTA = *Centro de Testagem e Aconselhamento* (Testing and Counseling Center); ^||^SAE = *Serviço de Atenção Especializada* (Specialized Care Service); ^¶^BHU = Basic Health Unit; **Pearson’s Chi-Square Test; ^††^Normality test using the Shapiro-Wilk test and evaluation of histograms, with subsequent use of Student’s or Wilcoxon’s t tests, parametric test and its non-parametric counterpart; ^‡‡^Mean (Standard Deviation); ^§§^CD4+ = Immune system cells (lymphocytes)

The best logistic regression analysis model consisted of five variables. In the adjusted analysis, it was verified that the older the age, the greater the chances of treatment abandonment (OR_adj_: 1.47; 95% CI: 1.07-2.13; p=0.024). Adolescents and young people who sometimes used condoms (OR_adj_: 0.22; 95% CI: 0.07-0.59; p=0.003) and those who had opportunistic infections (OR: 0.31; 95% CI: 0.10-0.90; p= 0.030) were less likely to abandon ART, which are considered protective factors. In the predictive logistic model using the ROC curve, overall accuracy was verified with an area of 0.63 (95% CI: 0.47-0.78), being statistically significant (p=0.001) with 83% sensitivity, which corresponds to the ability to classify the model as with good predictive capacity (true positive) (Table4).

**Table 4 -:** Logistic regression and ROC curve* according to the variables with statistical significance for ART abandonment among adolescents and young people living with HIV/AIDS in the 15^th^ Health Region. Maringá, PR, Brazil, 2022

Estimation of the Logistic Regression Model Parameters					
Variables	OR^†^	95% CI^‡^	OR_adj_^§^	95% CI^‡^	p value^||^
**Age at last consultation**					
Mean of 22.8	1.27	[0.99-1.71]	1.47	[1.07-2.13]	0.024
**Distance to the service (in km** ^¶^ **)**					
Mean of 17.3	1.02	[0.99-1.04]	1.02	[0.99-1.05]	0.101
**Condom use**					
Never	1	-	1	-	-
Sometimes	0.23	[0.08-0.59]	0.22	[0.07-0.59]	0.003
Always	0.59	[0.14-2.02]	0.41	[0.08-1.56]	0.217
**Opportunistic infections**					
No	1	-	1	-	-
Yes	0.42	[0.17-1.09]	0.31	[0.10-0.90]	0.030
Adequacy of the Predictive Logistic Model by the ROC Curve*					
Overall Accuracy	0.63	[0.47-0.78]			0.001
Sensitivity	0.83				
Specificity	0.60				

^*^ROC = Receiver Operating Characteristic Curve; ^†^OR = Unadjusted Odds Ratio; ^‡^CI = Confidence Interval; ^§^OR_adj_ = Adjusted Odds Ratio; ^||^Significant when <0.05; ^¶^km = Kilometer

## Discussion

By identifying the factors associated with ART abandonment among adolescents and young people living with HIV/AIDS during the COVID-19 pandemic, it was observed that age close to the mean of 22.8 years old at the last consultation was a factor that indicated greater chances of ART abandonment. Living more than 17.3 km away from the service remained as a control variable for the others, considering its influence on health care. Sporadic condom use and having opportunistic infections were protective factors for ART abandonment. These findings allow for reflections on the implications that transpose the diagnosis and clinical monitoring of adolescents and young people living with HIV/AIDS during the COVID-19 pandemic, in interface with individual, lifestyle and infection-related issues.

Considering that the study started from a retrospective analysis, the information bias as a result of deficiencies in the records due to the use of secondary sources is acknowledged as a limitation. However, in situations with lack of crucial information for case and control classification, the medical records were excluded.

Among the sociodemographic characteristics, age close to the mean of 22.8 years old at the last consultation within the monitoring period presented am increased chance of ART abandonment. Thus, the fact of being in a transition period from young to adult care is configured as an element that predisposes to the interruption of outpatient follow-up, even more during a pandemic that restricts social contact and access to health services due to health emergency focused on COVID-19^(17)^.

Corroborating the findings, a study carried out in Uganda with adolescents and young people living with HIV, selected in specialized clinics, showed that care transition generally occurs between 22 and 24 years of age, as cognitive, sexual, psychological and physical maturity can influence this process^(18)^. A retrospective cohort study, carried out with adolescents and young people in Tanzania, also identified that being aged between 20 and 24 years old was associated with reduced adherence, showing that one out of three young individuals abandon ART^(19)^.

Even in this care transition context, given the weakness of the immune system since diagnosis, together with the pandemic scenario, there were many challenges and changes, both of a psychological and social nature and biological. This is a context that portrayed a higher risk of infections and complications related to COVID-19 and AIDS progression, even more in people who do not undergo treatment^(20)^.

It is noted that a weak transition can put patients at risk due to the stigma faced, consequently causing monitoring discontinuity, reflecting in the decrease in adherence to care measures and ART. Thus, there is an increase in resistance to medications and a decrease in immunity, thus leading to morbidity due to opportunistic infections and virus transmission risk, especially when adolescents and young people are sexually active^(17-18)^.

So that there is no abandonment of care measures and ART, it is important that care transition is successful, especially when experiencing a pandemic period in which everyday life is permeated by challenges. However, it is known that many adolescents and young people fail to participate in health services during this life phase because they are unaware of the true need for treatment, thus, the transition process is still a difficulty in the HIV care cascade^(21)^.

The “distance to the service in km” variable, when living more than 17.3 km from the service, remained as a control for the other variables, being associated with ART abandonment, which corroborates another study which showed that living more than 5 km away from the health unit was a predictor of non-adherence to ART^(22)^.

The interference of geographic access, distance and commute from the patient’s home to the health service is a major obstacle, especially as a result of the service offered to people living with HIV/AIDS being centralized in a single physical space^(23)^. It is important to consider that, along the geographic route, some subjects need a longer period of time to commute to the specialized care service and, consequently, face greater accessibility obstacles for care and access to ART^(9)^.

It is noted that access to the specialized health care service is an influential factor in adherence to HIV/AIDS care measures and ART, mainly due to its chronicity. As a result, lack of integration with other health services, especially those that are part of Primary Care, associated with the centralization of specialized care, may favor the fragmentation in the development of health promotion and prevention actions and strategies^(23)^.

The viral suppression probability is related to adherence to consultations and ART, thus improving health results in the long term. However, during the pandemic period, adolescents and young people were vulnerable to interruptions in monitoring and treating the disease. Social isolation and/or distancing was the main preventive measure during the pandemic; therefore, the interactions with health professionals from the specialized care service, the half-yearly monitoring of CD4 and Viral Load and access to medications for the treatment of HIV and opportunistic diseases were impaired, as many of the patients use public transportation to commute to the health service and, afraid of being contaminated with COVID-19, they ended up not adhering to the treatment^(20,24)^.

With regard to the epidemiological and behavioral characteristics, in the current study it was identified that occasional condom use is a protective factor for treatment abandonment among adolescents and young people living with HIV/AIDS. A study developed in Uganda identified high rates of young people on ART who are sexually active and occasionally use condoms^(25)^. A survey conducted online with 148 people living with HIV/AIDS during the pandemic indicated that 12.2% reduced condom use during sexual intercourse^(26)^.

The patients who do use condoms, even if occasionally, are more likely to adhere to ART. However, there is a need to encourage condom use and adherence to ART since, in cases of inconsistent condom use among patients with HIV and risk of non-adherence to ART, the number of HIV superinfection cases (infection of an HIV-positive individual with another strain of the virus) and resistance to medications can be increased, in addition to the spread of strains resistant to antiretrovirals to their steady or casual partners^(27-29)^.

In this sense, health professionals need to further strengthen the self-care actions^(25,30)^, encouraging combined prevention, as adolescents and young people are a priority population for access to biomedical interventions: distribution of external and internal condoms, lubricants, use of antiretrovirals providing access to Treatment for All (TfA), Post-Exposure Prophylaxis (PEP) and Pre-Exposure Prophylaxis (PrEP); behavioral interventions: encouraging condom use, testing, adherence to biomedical interventions, counseling on HIV/AIDS, harm reduction for people who use alcohol and other illicit drugs, as well as communication and health education among peers; and, finally, structural interventions: actions aimed at sociocultural factors that directly influence the vulnerability of individuals or social groups to HIV; it is highlighted that all interventions can be used together, in order to reduce HIV transmission among peers^(7,30)^.

It is believed that ART abandonment during the pandemic can also be a reflection of people who still do not fully understand the importance of taking care of themselves and others^(25)^. However, it is important to consider that, in the context of the study during COVID-19, many patients ended up becoming undetectable (an HIV+ person with undetectable viral load who does not transmit HIV sexually) and, in some cases, their partners used PrEP; therefore, they did not always use condoms.

In relation to the characteristics of diagnosis and monitoring, the presence of one or more opportunistic infections proved to be a protective factor for abandonment. The patients who develop AIDS present low CD4 counts; therefore, they have reduced immunity and, consequently, become sicker and prone to opportunistic infections. A study carried out in Tanzania evidenced that symptomatic patients or those who perceive some health risk are more likely to adhere to ART in order to improve their health condition^(19)^.

The health service, locus to this research, underwent substantial changes during the pandemic period, such as a reduction in the number of consultations per day and medical prescriptions with longer times to acquire the medications in order to avoid crowding and contamination by COVID-19, which ended up limiting the activities already scheduled. In a study, it was observed that municipalities with more demand for COVID-19 have an estimate of deaths due to HIV in five years with a 10% increase, when compared to non-pandemic periods^(31)^.

In Brazil, the fight against HIV has been considered exemplary; however, many people living with HIV still do not have access to the treatment and, during the last two years, with the repercussions of COVID-19, progress against HIV has faltered, so many lives are at risk. Even if there is a public offer to fight against HIV/AIDS, this does not mean that all people will have access to the service, as social inequality, discrimination and stigma are present in the Brazilian territory. In the face of social diversity, there are still many poor and marginalized communities, especially the most vulnerable population, consequently reflecting on a significant impact on access to ART during the pandemic^(32)^.

The study has internal validity due to the quality of the evidence found. Although the study meets the objective proposed and the hypothesis, it was carried out in a local and not a national reality, consequently precluding generalization of the findings to other contexts. This fact suggests conducting research studies with larger populations.

However, within the Health and Nursing scope, this study provided theoretical and operational knowledge through the identification of factors associated with ART abandonment among adolescents and young people during a pandemic period, which can support public health policies for planning and management the care provided to adolescents and young people living with HIV/AIDS. In addition, investments should be made in social support for these patients, their family members and the health team, especially the Nursing team, as work should involve everyone who knows about the serology of adolescents and young people in order to improve quality of life and survival based on adherence to ART.

## Conclusion

Age close to 23 years old at the last consultation was associated with antiretroviral therapy abandonment. The presence of opportunistic infections and sporadic condom use are determining factors for ART abandonment during the COVID-19 pandemic, evidencing that sociodemographic, epidemiological and behavioral characteristics influence treatment cessation.

Therefore, this and future studies allow listing possibilities for changes in the scenario of the care provided to adolescents and young people in coping with HIV/AIDS, as the reasons for abandoning and following ART were elucidated. This enables a review of public policies and the effective performance of nurses in the elaboration of a care plan consistent with the needs and demands presented by the subjects who live in this context and are prone to treatment abandonment.
